# Iberverin Downregulates GPX4 and SLC7A11 to Induce Ferroptotic Cell Death in Hepatocellular Carcinoma Cells

**DOI:** 10.3390/biom14111407

**Published:** 2024-11-05

**Authors:** Haoying Yang, Bolei Dai, Liangjie Chen, Yingping Li, Xiaorui Jin, Chengchang Gao, Linfen Han, Xueli Bian

**Affiliations:** 1 The MOE Basic Research and Innovation Center for the Targeted Therapeutics of Solid Tumors, School of Basic Medical Sciences, Jiangxi Medical College, Nanchang University, Nanchang 330031, China; yanghaoying2023@163.com (H.Y.); daibolei@ncu.edu.cn (B.D.); chenliangjie9992@163.com (L.C.); jinxiaorui@ncu.edu.cn (X.J.); chengchanggao@163.com (C.G.); hanlinfen163@163.com (L.H.); 2 Shanxi Academy of Advanced Research and Innovation, Taiyuan 030032, China; liyingping1990@163.com

**Keywords:** iberverin, HCC, ferroptosis, GPX4, SLC7A11

## Abstract

Ferroptosis, a recently elucidated style of regulated cell death, has emerged as a significant area of investigation in cancer biology. Natural active compounds that have anti-cancer effects are promising candidates for cancer prevention. Iberverin, a natural compound derived from *Brassica oleracea* var. *capitata*, has been shown to exert anti-tumor activities in some cancers. However, its role in hepatocellular carcinoma (HCC) cells and the molecular mechanisms are still poorly understood. In this study, we proved that iberverin can induce intracellular reactive oxygen species (ROS) generation to inhibit cell proliferation and initiate ferroptotic cell death in HCC cells, which can be eradicated by the ferroptosis inhibitor ferrostatin-1 (Fer-1) or deferoxamine mesylate (DFO) and ROS scavenger (GSH or NAC). Mechanistically, iberverin treatment can simultaneously downregulate *SLC7A11* mRNA level and degrade GPX4 through the ubiquitination pathway, leading to lipid peroxidation and ferroptotic cell death in HCC cells. Significantly, a low dose of iberverin can remarkably increase the sensitivity of HCC cells to ferroptosis induced by canonical ferroptosis inducers RSL3 and imidazole ketone erastin (IKE). This study uncovers a critical function of iberverin in preventing HCC through ferroptosis and provides a promising strategy for HCC treatment either via iberverin alone or in combination with canonical ferroptosis inducers in the future.

## 1. Introduction

Hepatocellular carcinoma (HCC) is a prevalent and aggressive form of primary liver cancer, accounting for most cases globally. It is the third most common malignancy contributing to cancer-related mortality among cancer patients. The incidence of HCC varies geographically, with a notably higher prevalence in Asia compared to other regions [1,2]. Given its aggressive nature and generally dismal prognosis, the identification of effective therapeutic strategies for HCC remains a formidable challenge, posing significant obstacles to improve patient outcomes [3]. Sorafenib is the primary drug utilized for HCC treatment in clinical practice; however, although it has shown good efficacy for HCC patients, most patients develop resistance in the late stage [4,5]. Therefore, the adoption of a comprehensive treatment strategy can effectively solve this dilemma. It is reported that natural active products have a great deal of anti-tumor activities with minimal side effects [6,7]. Therefore, finding new natural products for the treatment of HCC patients and understanding their mechanisms are of great significance for the treatment and prevention of HCC.

Ferroptosis is a form of programmed cell death, marked by the accumulation of iron and reactive oxygen species (ROS) and subsequent lipid peroxidation [3,8,9]. Lipid peroxides serve as key intermediates in lipid peroxidation, with glutathione peroxidase 4 (GPX4) catalyzing the conversion of lipid peroxides into less harmful alcohols [10,11]. This enzymatic activity is important for maintaining cellular lipid homeostasis and mitigating the adverse effects of reactive oxygen species (ROS) on cellular membranes [12,13,14]. The activity of GPX4 is crucial for preventing the buildup of harmful ROS that can compromise cell integrity. The system Xc- is a sodium-independent transporter that imports cystine in exchange for the export of intracellular glutamate, comprising a heterodimer formed by the heavy chain subunit, solute carrier family 3 member 2 (SLC3A2), and the light chain subunit, solute carrier family 7 member 11 (SLC7A11) [15,16,17]. Additionally, SLC7A11 facilitates the import of cystine into cells, which is vital for glutathione (GSH) biosynthesis and antioxidant defense [15]. Ferroptosis is a distinct form of cellular death that diverges from other death modalities, such as autophagic cell death and apoptosis, in terms of functional and morphological characteristics [1,18,19]. Notable features of ferroptosis include increased mitochondrial membrane density, pronounced mitochondrial contraction, and reduced or absent mitochondrial cristae [20,21]. Class I ferroptosis inducers (FINs), including erastin, initiate iron-dependent cell death by reducing GSH through the inhibition of cystine importer SLC7A11 [15]. In contrast, class II FINs, such as Ras-selective lethal 3 (RSL3), induce ferroptosis by directly inhibiting GPX4 [22]. Compounds such as RSL3 and IKE (an erastin analog) have been shown to significantly reduce the viability of HCC cells [23,24]. Interestingly, cancer cells are prone to ferroptosis induction due to their metabolic reprogramming and the resultant accumulation of ROS [25,26,27,28,29]. Therefore, targeting cancer cell ferroptosis may be a potential cancer treatment strategy. HCC cells are not only challenging to treat but also come with numerous drawbacks and side effects associated with current therapeutic drugs [30,31,32,33]. Therefore, it is urgent to discover new compounds that either induce ferroptosis or increase ferroptosis sensitivity, especially natural compounds, for the treatment of HCC with minimal side effects.

Iberverin, a natural compound extracted from *Brassica oleracea* var. *capitata*, is a biologically active natural compound that has been reported to be an anti-tumor compound [34]. Iberverin can inhibit the proliferation and induce apoptosis of non-small cell lung cancer A549 cells in a time- and dose-dependent manner [35]. A caspase activity assay revealed that, compared with other fractions extracted from *Brassica oleracea* var. *capitata*, iberverin can mildly activate caspase-3/8/9 [36]. In HCC cells, iberverin treatment can inhibit cancer cell migration, invasion, and cell proliferation dependent on the G2/M cell cycle arrest induced by DNA damage [34]. Furthermore, iberverin can also induce HCC cell apoptosis and inhibit HCC tumorigenicity in vivo without systematic toxicity [34]. However, the detailed mechanism of its anti-tumor activity and the specific targets of iberverin are largely unknown.

In this study, we revealed that the natural compound iberverin downregulates the *SLC7A11* mRNA level and promotes GPX4 degradation through the ubiquitination pathway, leading to the inhibition of cell proliferation and ferroptotic cell death in HCC cells. Importantly, the combination of low dose of iberverin with canonical ferroptosis inducers RSL3 and imidazole ketone erastin (IKE) can synergistically induce HCC cell death.

## 2. Materials and Methods

### 2.1. Cell Lines and Cell Culture

The hepatocellular carcinoma cell lines HLE and HCCLM3 were purchased from the Cell Bank of the Chinese Academy of Sciences. All the cells were cultured in DMEM Medium (Solarbio, Cat# 11965, Beijing, China) supplemented with 10% fetal bovine serum (Excell Bio, FSP500, Shanghai, China) and maintained at 37 °C in a humidified atmosphere containing 5% CO_2_. After 1 to 2 days of culture, the culture medium was replaced with fresh DMEM. The cells were harvested at approximately 80~90% confluence during the logarithmic growth phase and were either utilized for subsequent experiments or passaged as needed.

### 2.2. Regents and Antibodies

The following reagents were utilized in this study: Iberverin (TargetMol, Cat# T7873, China); N-acetyl-L-cysteine (NAC, MCE, Cat# HY-B0215, Shanghai, China); DCFH-DA (MCE, Cat# HY-D0940, Shanghai, China); L-Glutathione reduced (GSH, MCE, Cat# HY-D0187, Shanghai, China); ferrostatin-1 (Fer-1, Abmole, Cat# M2698, Shanghai, China); Deferoxamine Mesylate (DFO, TargetMol, Cat# T1637, Boston, MA, USA); DCFH-DA (MCE, Cat# HY-D0940, Shanghai, China); Imidazole ketone erastin (IKE, TargetMol, Cat# T5523, Boston, MA, USA); TRIzol Up reagent (TransGen, Cat# ET111-01-V2, Beijing, China); Cycloheximide (CHX, MCE, Cat# HY-12320, Shanghai, China); M5 HiPer One-Step RT-PCR Kit (Mei5bio, Cat# MF051-01, Beijing, China); Z-Leu-Leu-Leu-CHO (MG132, Biovision, Cat# 1791-5, Milpitas, CA, USA); ABI QuantStudio 7 Flex System equipped with SYBR Green PCR kit (TransGen, Cat# AQ601-02, Beijing, China); RSL3 (CSNpharm, Cat# CSN17581, Chicago, IL, USA); PMSF (Wuhan Dingguo Biotechnology, Cat# 329-98-6, Wuhan, China); paraformaldehyde (Solarbio, Cat# P1110, Beijing, China); M5 Super Plus qPCR RT Kit with gDNA remover (Mei5bio, Cat# MF166-plus-T, Beijing, China); and C11-BODIPY581/591 (MCE, Cat# HY-D1691, Shanghai, China).

The antibodies employed in this investigation included: GPX4 (Proteintech, Cat# 67763-1-Ig, Wuhan, China, diluted 1:2000); Vinculin (Santa Cruz, Cat# sc-73614, Dallas, TX, USA, diluted 1:30,000); SLC7A11 (Proteintech, Cat# 18790-1-AP, Wuhan, China, diluted 1:3000); HA (Santa Cruz, Cat# sc-7392, Dallas, TX, USA, used at 1:5000); ACSL4 (Proteintech, Cat# 22401-1-AP, Wuhan, China, diluted 1:6000); Flag (Sigma, Cat# 66008-3Ig, Livonia, MI, USA, used at 1:5000); and HRP conjugated goat anti-rabbit (Thermo Fisher Scientific, Cat# 31460, Waltham, MA, USA, used at 1:10,000) or goat anti-mouse secondary antibodies (Thermo Fisher Scientific, Cat# 31437, Waltham, MA, USA, used at 1:10,000).

### 2.3. Western Blot

The cells were lysed with a cell lysis buffer containing 20 mM Tris, 1 mM Na_2_EDTA, 150 mM NaCl, 1 mM EGTA, 2.5 mM Sodium pyrophosphate, and 1% (*v*/*v*) Triton X-100, with the pH adjusted to 7.5 using hydrochloric acid. Additionally, 1% PMSF was added to the buffer. The lysis was carried out at 4 °C for 40 min. The resulting lysates were transferred to 1.5 mL Eppendorf tubes and subsequently centrifuged at 13,200× *g* for 15 min at 4 °C. After centrifugation, the supernatant was collected into a new 1.5 mL tube and mixed with an equal volume of 2X SDS loading buffer, followed by boiling at 100 °C for 5 min. The proteins were separated by SDS-PAGE and then transferred to polyvinylidene fluoride (PVDF) membranes (Millipore, Cat# 03010040001, Burlington, MA, USA). Then, the PVDF membrane was placed in 5% non-fat dry milk (Solarbio, Cat# D8340, Beijing, China) and blocked at room temperature for 1 h, followed by incubation with the appropriate primary and secondary antibodies. After washing 3 times with Tris-Buffered Saline with Tween-20, protein detection was facilitated via an enhanced ChemiLuminescence (ECL) detection kit (Meilunbio, Cat# MA0186, Dalian, China).

### 2.4. Cell Viability Assay

The cell viability was assessed via the Cell Counting Kit-8 (CCK-8). Briefly, the cells were harvested from the culture plates, resuspended, and counted. A total of 200 μL of cell suspension, containing 6000 cells per well, was seeded into a 96-well plate, and incubated under standardized conditions. Following the appropriate incubation period, 20 μL of CCK-8 solution was added to each well and the plate was incubated for 1 h. The cell viability was calculated by measuring absorbance at 450 nm through a microplate reader.

### 2.5. Quantitative Real-Time PCR (RT-qPCR)

The total RNA was extracted from cultured cells via TRIzol reagent (Invitrogen, Cat# 15596-026) in accordance with the manufacturer’s protocol. The integrity, purity, and concentration of the RNA were assessed via a NanoDrop 2000c spectrophotometer (Thermo Fisher Scientific). The genomic DNA was subsequently removed via an M5 Super Plus qPCR RT Kit (Mei5bio, Cat# MF166-plus-T). The total RNA was subsequently reverse transcribed into complementary DNA (cDNA) utilizing the M5 HiPer One-Step RT-PCR kit (Mei5bio, Cat# MF051-01). A real-time quantitative PCR was conducted in a 20 µL reaction volume with three biological replicates per sample. A SYBR Green PCR kit (TransGen, Cat# AQ601-02, China) was employed for amplification via, subsequently, an ABI QuantStudio 7 Flex system for detection. The relative gene expression levels were quantified using the comparative Ct method, where the Ct values were normalized to that of β-actin. The real-time quantitative PCR primers were used as follows:

SLC7A11 (human) forward: 5′-GGTCCATTACCAGCTTTTGTACG-3′, SLC7A11 (human) reverse: 5′-AATGTAGCGTCCAAATGCCAG-3′;
GPX4 (human) forward: 5′- GAGGCAAGACCGAAGTAAACTAC-3′; GPX4 (human) reverse: 5′-CCGAACTGGTTACACGGGAA-3′;
β-Actin (human) forward: 5′-GTCACCAACTGGGACGACA-3′; β-Actin (human) reverse: 5′-CACAGCCTGGATAGCAACG-3′.

### 2.6. Cell Death Analysis

The cell death rate was assayed using propidium iodide (PI) staining followed by flow cytometry analyses. In brief, the floating and attached cells were collected and stained with PI (10 μg/mL) at room temperature for 15 min under light-proof conditions. After staining, the cells were collected and washed twice with PBS and then resuspended in 500 μL of PBS for the flow cytometry analysis in the channel of PE. The data were analyzed via FlowJo 10 software.

### 2.7. Detection of Intracellular ROS and Lipid ROS Levels

Flow cytometry was used to detect the intracellular ROS and lipid ROS levels according to the manufacturer’s instructions. The cells were first inoculated in 12-well plates and incubated for the appropriate time. The culture medium was replaced with PBS containing the ROS probe DCFH-DA (MCE, Cat# HY-D0940, China) or the lipid ROS probe CII-BODIPY581/591 (MCE, Cat# HYD1691, China) and the cells were subsequently incubated at 37 °C for 30 min. After incubation, the cells were collected and resuspended in 500 μL PBS for the cytometric analysis in the channel of FITC. The data were analyzed via FlowJo 10 software.

### 2.8. Colony Formation Assay

In this experiment, colony formation was used to assess the cell viability. First, the cells were seeded into six-well plates and incubated with DMEM containing 10% fetal bovine serum (FBS) and 5% CO_2_ at 37 °C for 36 h. Following incubation, the medium was carefully removed, and the cells were washed three times with PBS. Paraformaldehyde (Solarbio, Cat# P1110, China) was then added into the plates to fix the cells for 15 min, after which the cells were washed twice with PBS. Subsequently, the cells were stained with crystal violet solution (0.1% crystal violet) for two minutes and finally photographed.

### 2.9. Statistical Analysis

All the statistical analyses were performed with GraphPad Prism 10 software, and all the quantitative data are expressed as the mean ± standard deviation (SD) of three or more independent experiments. Comparisons between two groups were analyzed using the Student’s *t* test, and comparisons among more than two groups were analyzed using one-way ANOVA (analysis of variance). Differences were considered statistically significant if *p* < 0.05.

## 3. Results

### 3.1. Iberverin Inhibits the Viability of HCC Cells

**Figure 1:**
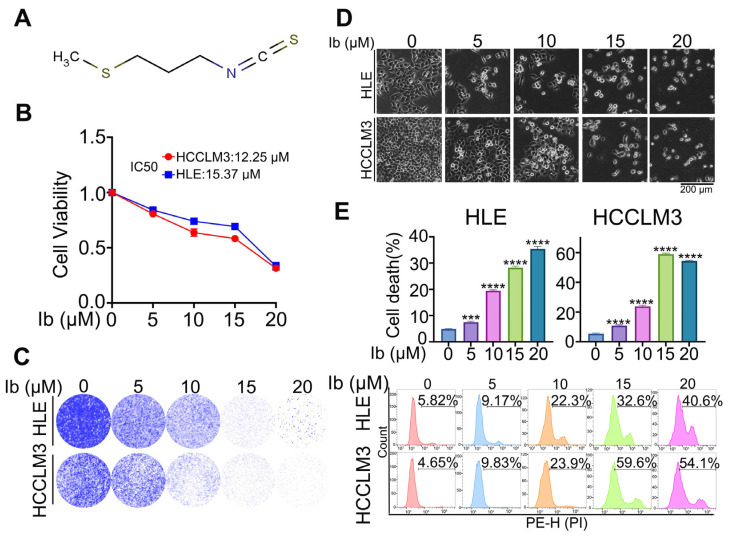
Iberverin exerts anti-tumor effects in HCC cells. (**A**) The molecular structure of iberverin. (**B**) HLE and HCCLM3 cells (5000 cells/well) were inoculated, seeded into 96-well plates, and treated with different doses of iberverin for 48 h. Cell viability was assayed using a CCK-8 kit, the absorbance at 450 nm was measured via a microplate reader, and IC50 was calculated. Data have been normalized to the iberverin = 0 μM group. (**C**–**E**) HLE and HCCLM3 cells were treated with the indicated concentrations of iberverin for 48 h. Cells were stained with crystal violet and photographed, and the images are shown in (**C**). The cells were photographed and images are shown in (**D**). Cells were harvested and stained with PI, and then a flow cytometric analysis was performed in the PE-H channel. The calculated cell death rate (top) and representative flow cytometric images (bottom) are shown in (**E**). (**B**,**E**) Data are represented as the mean ± SD (*n* = 3); *** *p* < 0.001, **** *p* < 0.0001 (one-way ANOVA). Ib: Iberverin.

Iberverin is a natural product isolated from *Brassica oleracea* var. *capitata* with a molecular weight of 147.26 (Figure 1A). To investigate its role in HCC cells, we treated HLE and HCCLM3 cells with various concentrations of iberverin for 48 h. The CCK-8 assay shows that iberverin treatment significantly inhibited HCC cell viability (Figure 1B). The colony formation assay also reveals that iberverin inhibited HCC cell’s colony formation ability in a dose-dependent manner (Figure 1C). Of note, the morphological analysis shows a marked increase in the proportion of floating HLE and HCCLM3 cells after treatment with iberverin (Figure 1D), implying that iberverin could induce HCC cell death. To prove it, HCC cells stimulated with different concentrations of iberverin were stained with PI and the flow cytometric analysis shows that iberverin treatment could significantly induce HLE and HCCLM3 cell death in a dose-dependent manner (Figure 1E). Collectively, these data demonstrate that iberverin exerts anti-tumor effects via cell viability inhibition and cell death induction in HCC cells.

### 3.2. ROS Is Responsible for the Anti-Tumor Effects of Iberverin in HCC Cells

**Figure 2:**
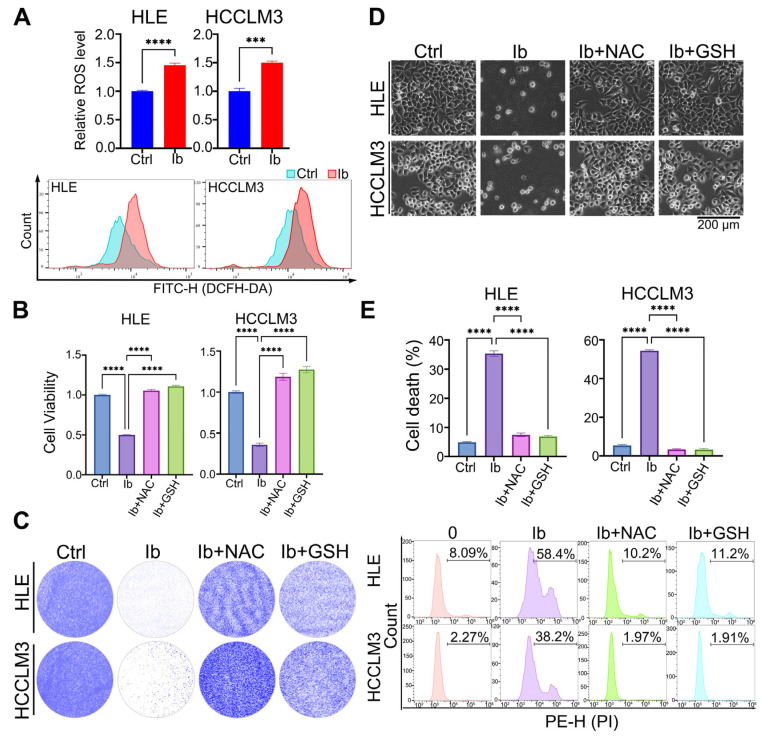
ROS induction is responsible for iberverin-induced HCC cell death. (**A**) HCC cells were divided into two groups: the control group (Ctrl), and the iberverin group (Ib). After 8 h of iberverin (20 μM) treatment, the cells were incubated with an ROS probe DCFH-DA (10 μM) for 30 min and collected for intracellular ROS level analyses with flow cytometry. The normalized ROS levels (top) and the representative flow cytometric images (bottom) are shown. (**B**–**E**) HLE and HCCLM3 cells (5000 cells/well) were seeded into a 96-well plate and treated with NAC (5 mM), GSH (5 mM), or iberverin (20 μM) for 48 h. (**B**) Cell viability was assayed via a CCK-8 kit and the absorbance at 450 nm was measured using a microplate reader. Data have been normalized to the control group. (**C**) HCC cells were stained with crystal violet and photographed, and the representative images are shown. (**D**) Cells were photographed and the representative images are shown. (**E**) Cells were collected and stained with PI, and then flow cytometric analysis was performed in the PE-H channel. The cell death rate (top) and representative flow cytometric images (bottom) are shown. (**A**,**B**,**E**) Data are presented as the mean ± SD (*n* = 3); *** *p* < 0.001, **** *p* < 0.0001 (unpaired Student’s *t* test for (**A**) and one-way ANOVA for (**B**,**E**)).

The above data showed that iberverin can function as an anti-tumor agent in HCC cells. Next, we set out to identify the detailed mechanism by which iberverin prevents HCC cell viability. It has been reported that iberverin increases ROS levels in HCC cells, but the exact mechanism remains unclear [34]. To determine whether ROS generation is responsible for the inhibition of HCC cells induced by iberverin, firstly we determined the intracellular ROS level in HLE and HCCLM3 cells with a DCFH-DA probe. The flow cytometric analyses reveal that iberverin treatment could significantly induce ROS production in HCC cells (Figure 2A); this finding is consistent with a previous report [34]. Secondly, we performed CCK8 cell proliferation assays with the addition of the antioxidants N-acetylcysteine (NAC) and glutathione (GSH) and found that the inhibition of cell proliferation caused by iberverin was greatly attenuated by NAC and GSH (Figure 2B). The results of the crystal violet staining experiments (Figure 2C) and the photographic experiments (Figure 2D) also show the same results, suggesting that the antioxidants could significantly weaken the anti-tumor effects of iberverin in HCC cells. Finally, consistently, the cell death analyses show that NAC or GSH treatment significantly inhibited the cell death rate induced by iberverin in HCC cells (Figure 2E). Taken together, these results demonstrate that iberverin can lead to an excessive ROS accumulation in HCC cells to inhibit HCC cell proliferation and induce cell death.

### 3.3. Iberverin Induces HCC Cell Ferroptosis

**Figure 3:**
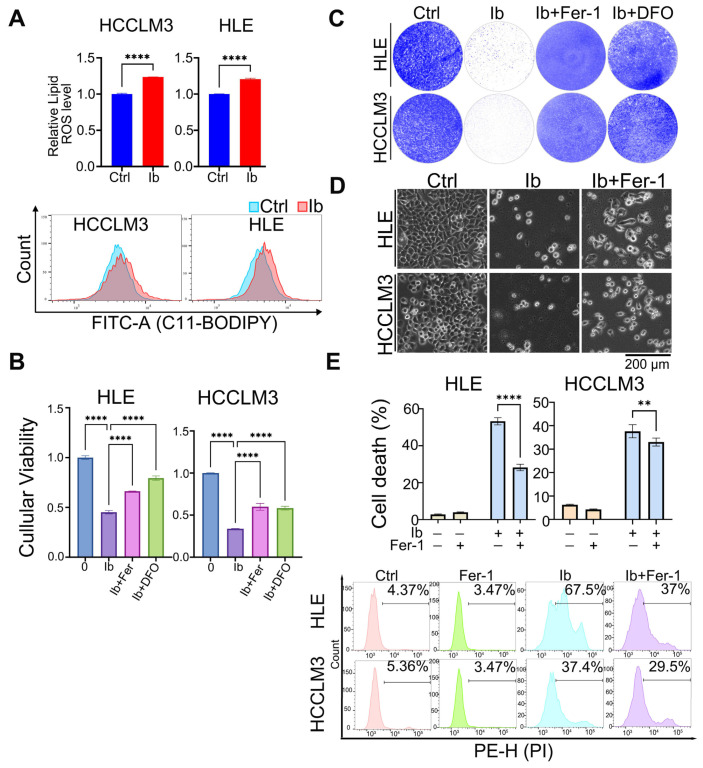
Iberverin induces HCC cell ferroptosis. (**A**) HCC cells treated with or without iberverin for 9 h were stained with 10 μM C11-BODIPY581/591 for lipid ROS detection with flow cytometry; the normalized lipid ROS levels (top) and the flow cytometric images (bottom) are shown. (**B**) Cells were treated with Fer-1 (2 μM), DFO (50 μM) and iberverin (20 μM) for 48 h. Cell viability was assayed with CCK-8 kit and the absorbance at 450 nm was measured using a microplate reader. Data have been normalized to the control group. (**C**) HCC cells were treated with Fer-1, DFO and iberverin and stained with crystal violet. (**D**) Cells were treated with Fer-1 (2 μM) and iberverin (20 μM) and photographed. (**E**) HCC cells treated with Fer-1 (2 μM) and iberverin (20 μM) for 48 h were collected for PI staining followed by flow cytometric analyses in the channel of PE-H. The cell death rate (**top**) and representative flow cytometric images are shown (**bottom**). (**A**,**B**,**E**) Data were represented as the mean ± SD (*n* = 3); (** *p* < 0.01; **** *p* < 0.0001) (unpaired Student’s *t* test for (**A**) and one-way ANOVA for (**B**,**E**)).

Since our results revealed that excessive ROS generation is responsible for the anti-tumor effects in HCC cells mediated by iberverin, next we focused on the specific manner in which iberverin inhibits HCC cell activities. Ferroptosis is a newly identified programmed cell death characterized by ROS- and iron-dependent lipid peroxidation and it was reported that cancer cells are prone to ferroptosis because of its high metabolic activity and demand of iron [37,38], making ferroptosis a potential strategy for cancer treatment [39,40]. Of note, there is no report about iberverin on ferroptosis regulation. Therefore, we wanted to know whether ferroptosis is involved in iberverin-induced HCC cell proliferation inhibition and cell death. Firstly, we examined the intracellular lipid ROS content by flow cytometry using the lipid ROS probe C11-BODIPY581/591, and the results reveal that iberverin treatment could significantly increase the lipid ROS in HCC cells (Figure 3A). Ferrostatin-1 (Fer-1), a potent ferroptosis inhibitor, can be used as an important criterion for the verification of ferroptosis. Deferoxamine Mesylate (DFO) is an iron chelator that binds to iron ions to form compounds that eliminate free iron ions. We used these two ferroptosis inhibitors here, and observed that the inhibitory effect of iberverin on cell proliferation was attenuated by the addition of Fer-1 and DFO (Figure 3B,C). In line with these findings, Fer-1 could significantly weaken the cell death rate induced by iberverin in HCC cells (Figure 3D,E). In summary, iberverin exerts anti-tumor effects in HCC cells via ferroptosis.

### 3.4. Low-Dose Iberverin Sensitizes HCC Cells to Ferroptosis

**Figure 4:**
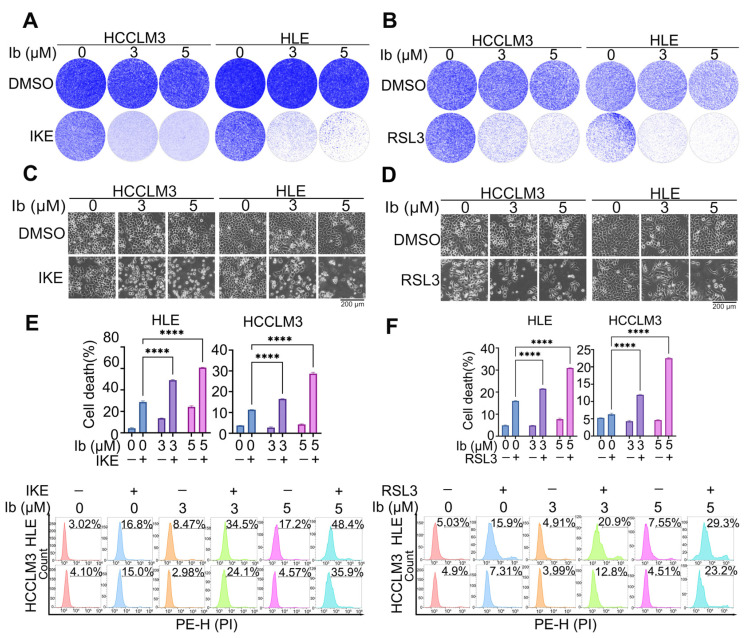
Low-dose iberverin sensitizes HCC cells to ferroptosis. (**A**–**F**) HCCLM3 and HLE cells were treated with the indicated doses of iberverin in the presence or absence of IKE (20 μM) (**A**,**C**) or RSL3 (5 μM) (**B**,**D**) were cultured for 48 h. (**A**,**B**) Cells were stained with crystal violet and finally photographed; the representative images are shown. (**C**,**D**) Cells were photographed and the representative images are shown. (**E**,**F**) Cells were collected and stained with PI, and then a flow cytometric analysis was performed in the PE-H channel; the calculated cell death rate (top) and representative flow cytometric images (bottom) are shown. (**E**,**F**) Data are represented as the mean ± SD (*n* = 3); **** *p* < 0.0001 (one-way ANOVA).

Our experiments have shown that high concentrations of iberverin can lead to ferroptosis in HCC cells, but higher concentrations can often mean greater side effects. Therefore, we aimed to explore whether the addition of low concentrations of iberverin in combination with ferroptosis inducers could enhance ferroptosis sensitivity in HCC cells. Two classical ferroptosis inducers IKE and RSL3 were used, and the colony formation assay shows that low concentrations of iberverin could significantly inhibit the cell proliferation induced by IKE or RSL3 in HLE and HCCLM3 cells (Figure 4A,B). Morphological observations reveal that the number of floating HLE and HCCLM3 cells was significantly increased by the addition of iberverin (Figure 4C,D), suggesting that low concentrations of iberverin do increase the susceptibility of HCC cells to ferroptosis. Finally, HCC cells were stained with PI, and the flow cytometry analyses show that there was no significant change in the cell death after the addition of low concentrations of iberverin, but the combination of RSL3 or IKE with iberverin significantly increased the cell death rate in HLE and HCCLM3 cells (Figure 4E,F). In conclusion, these data demonstrate that low concentrations of iberverin sensitize HCC cells to ferroptosis, underscoring the potential therapeutic strategy for HCC treatment by using a low concentration of iberverin in combination with classical ferroptosis inducers.

### 3.5. Iberverin Downregulates GPX4 and SLC7A11 Expression

**Figure 5:**
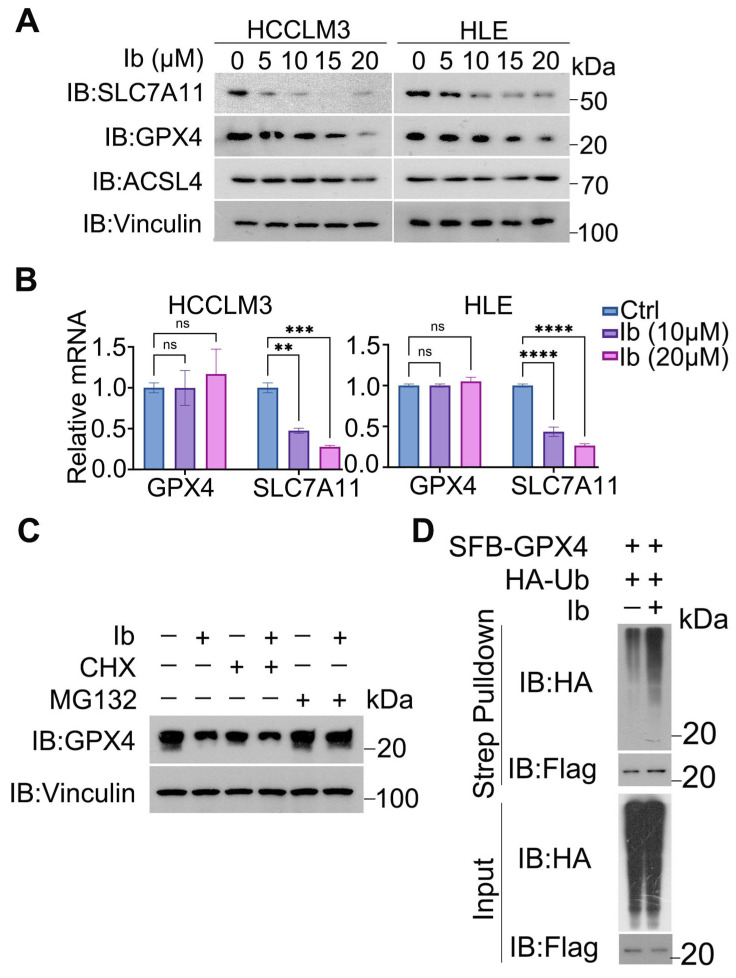
GPX4 and SLC7A11 were downregulated by iberverin. (**A**) Western blotting was performed to detect the protein expression levels of GPX4, SLC7A11, and ACSL4 in HCC cells treated with different concentrations of iberverin, with vinculin as a loading control. Original images can be found in Figure S1. (**B**) RT-qPCR detection of the mRNA levels of *GPX4* and *SLC7A11* in HCC cells treated with different concentrations of iberverin. (**B**,**C**) The data are presented as the means ± SD (*n* = 3); ns: not significant, (** *p* < 0.01, *** *p* < 0.001, **** *p* < 0.0001) (one-way ANOVA). (**C**) HLE cells were pre-treated with CHX (100 µg/mL) or MG132 (10 μM) for 2 h, followed by the addition of iberverin (20 μM) for 8 h. Protein samples were collected and analyzed by Western Blot. Original images can be found in Figure S2. (**D**) HLE cells were transfected with the indicated plasmids for 13 h and then treated with or without 20 µM iberverin for 10 h. Afterwards, the cells were lysed out and immunoprecipitated with an anti-flag antibody, followed by immunoblotting with the indicated antibodies. Strep: Streptavidin. Original images can be found in Figure S3.

Ferroptosis is tightly regulated by a series of ferroptosis-related proteins, such as GPX4, SLC7A11 and acyl-CoA synthetase long-chain family 4 (ACSL4) [19]. To determine whether ferroptosis-related proteins are regulated by iberverin, HCCLM3 and HLE cells were treated with different concentrations of iberverin. Western blot analyses show that SLC7A11 and GPX4 protein expressions were downregulated and ASCL4 protein expression presented no obvious change (Figure 5A). To ascertain the detailed regulatory mechanism, we measured the mRNA levels of *GPX4* and *SLC7A11* by real-time quantitative PCR. The results showed no significant change in the mRNA level of *GPX4* whereas the *SLC7A11* mRNA level gradually decreased with the increasing iberverin concentrations. It is suggested that GPX4 could not be regulated by iberverin at the mRNA level and iberverin may have affected the transcriptional activity of *SLC7A11* (Figure 5B). Then, we investigated the impact of iberverin on GPX4 protein levels by introducing CHX (an inhibitor of intracellular protein synthesis) and MG132 (a proteasome inhibitor). It was found that the protein content of GPX4 was reduced by the addition of CHX, which was further decreased upon the addition of iberverin (Figure 5C). Interestingly, pretreatment with MG132 eradicated the downregulation of the GPX4 protein level induced by iberverin, suggesting that iberverin may regulate GPX4 expression through post-translational modification (Figure 5C). To investigate the pathway through which iberverin regulates GPX4, we examined the ubiquitination level of GPX4. It was found that the ubiquitination of GPX4 was enhanced by the addition of iberverin (Figure 5D), suggesting that iberverin degraded GPX4 through the ubiquitin—proteasomal pathway. In conclusion, these results demonstrate that iberverin downregulates GPX4 and SLC7A11 protein levels to promote ferroptosis in HCC cells.

## 4. Discussion

Cell death is an indispensable part of the cellular life process and an important means of self-regulation for living organisms. Ferroptosis is a mode of cell death caused by the disruption of intracellular metabolic pathways leading to an excessive accumulation of lipid peroxides, and is closely related to intracellular iron metabolism and lipid homeostasis [14]. Tumor cells rely on high metabolic activities and iron levels to promote their sustained cell proliferation and growth, which makes them prone to ferroptosis. Therefore, targeting ferroptosis is a promising cancer treatment strategy.

**Figure 6:**
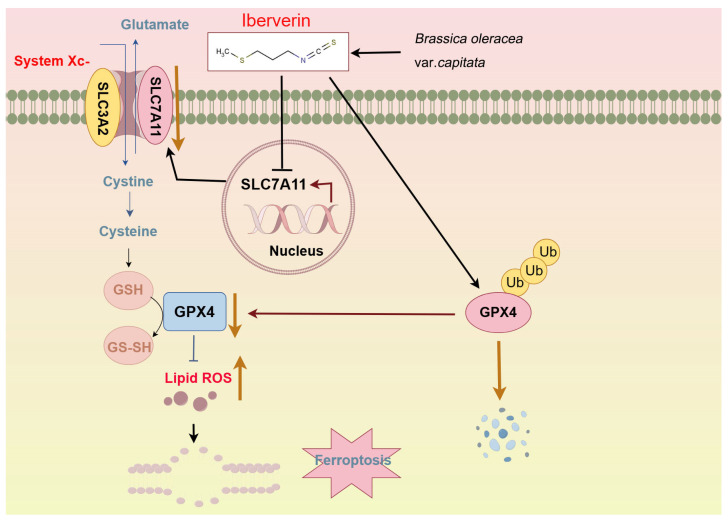
Working model of the proposed mechanism by which iberverin exerts anti-tumor effects in HCC cells via ferroptosis. The figure was created by Figdraw (www.figdraw.com).

Iberverin is a major isothiocyanate (ITC) isolated from *Brassica oleracea* var. *capitata*, and it was reported that iberverin inhibits tumor cell proliferation, migration, and invasion and induces apoptosis [34]. In addition, xenograft tumor experiments further confirmed the anti-tumor effects of iberverin in HCC in vivo without significant toxicity [34]. However, the detailed molecular mechanism by which iberverin functions as an anti-tumor agent in tumor cells, especially in HCC cells, and its association with ferroptosis are largely unknown. Our study demonstrates that iberverin can function as an anti-tumor agent by inhibiting cell proliferation and inducing ferroptotic cell death in HCC cells. Importantly, a low dose of iberverin can synergistically enhance ferroptotic cell death mediated by RSL3 and IKE. Mechanistically, iberverin treatment downregulates the *SLC7A11* mRNA level and promotes GPX4 ubiquitin—proteasome degradation, resulting in the downregulation of the ferroptosis defense proteins GPX4 and SLC7A11, eventually leading to excessive lipid peroxidation and ferroptosis in HCC cells (Figure 6).

Although our study reveals the molecular mechanism of iberverin on the regulation of GPX4 and SLC7A11 to influence ferroptosis in HCC cells, there are also some unanswered scientific questions. For example, does iberverin inhibit the mRNA stability or transcription of *SLC7A11*? If the transcription factor ATF4 is the main regulator for SLC7A11 transcription [41,42], does iberverin directly interact with ATF4 to inhibit its transcriptional activity? If GPX4 is regulated by a series of E3 ubiquitin enzymes or deubiquitinases [43,44,45], can iberverin directly interact with GPX4 or their regulators to regulate its stability? These scientific questions are all very important for our understanding of the role of iberverin in regulating HCC cell ferroptosis and remain to be explored in the future.

## 5. Conclusions

Our study shows that the natural product iberverin can simultaneously downregulate SLC7A11 and GPX4 expression to induce ferroptosis in HCC cells. Importantly, low concentrations of iberverin can increase the sensitivity of HCC cells to classical ferroptosis inducers, highlighting the potential of clinical HCC treatment by combining iberverin with classical ferroptosis inducers in the future.

## Data Availability

The data presented in this study are available upon request from the corresponding author.

## Supplementary Materials

The following supporting information can be downloaded at: https://www.mdpi.com/article/10.3390/biom14111407/s1, Figure S1: Original images of Figure 5A; Figure S2: Original images of Figure 5C; Figure S3: Original images of Figure 5D.
