# Supplementary material for: Iberverin Downregulates GPX4 and SLC7A11 to Induce Ferroptotic Cell Death in Hepatocellular Carcinoma Cells
Source: Biomolecules. 2024 Nov 5;14(11):1407. doi: 10.3390/biom14111407 (PMC11592392; doi:10.3390/biom14111407)
Supplement: Supplementary file 1 [file biomolecules-14-01407-s001.zip › biomolecules-3273442-supplementary.pdf]

Supplementary Materials for

Iberverin Downregulates GPX4 and SLC7A11 to Induce Ferroptotic Cell Death in Hepatocellular Carcinoma Cells

Haoying Yang [1,†], Bolei Dai [1,†], Liangjie Chen [1], Yingping Li [2], Xiaorui Jin [1], Chengchang Gao [1], Linfen Han [1] and Xueli Bian [1,*]

[*]Correspondence: bianxueli@ncu.edu.cn

This PDF file includes:

Original western blot images

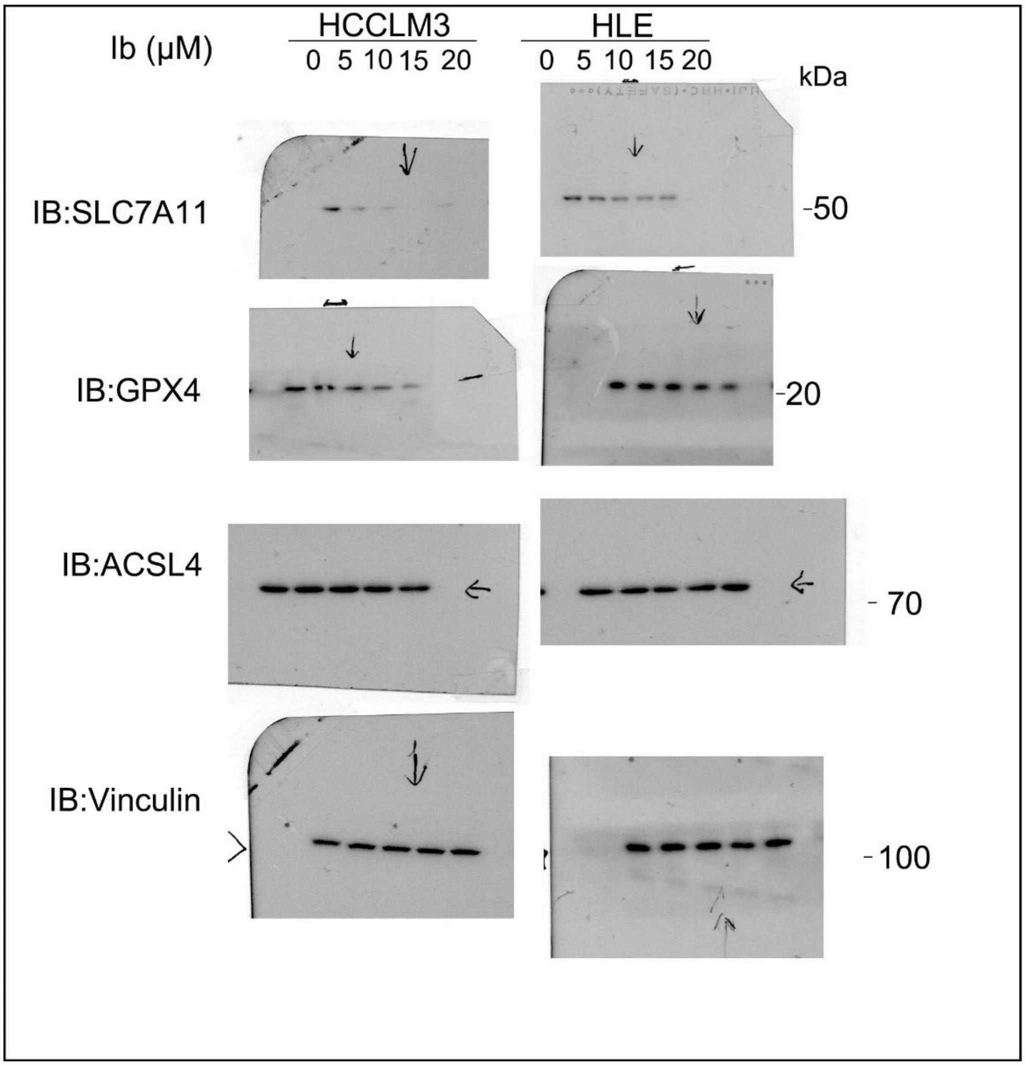

Figure S1. Original images of Figure 5A.

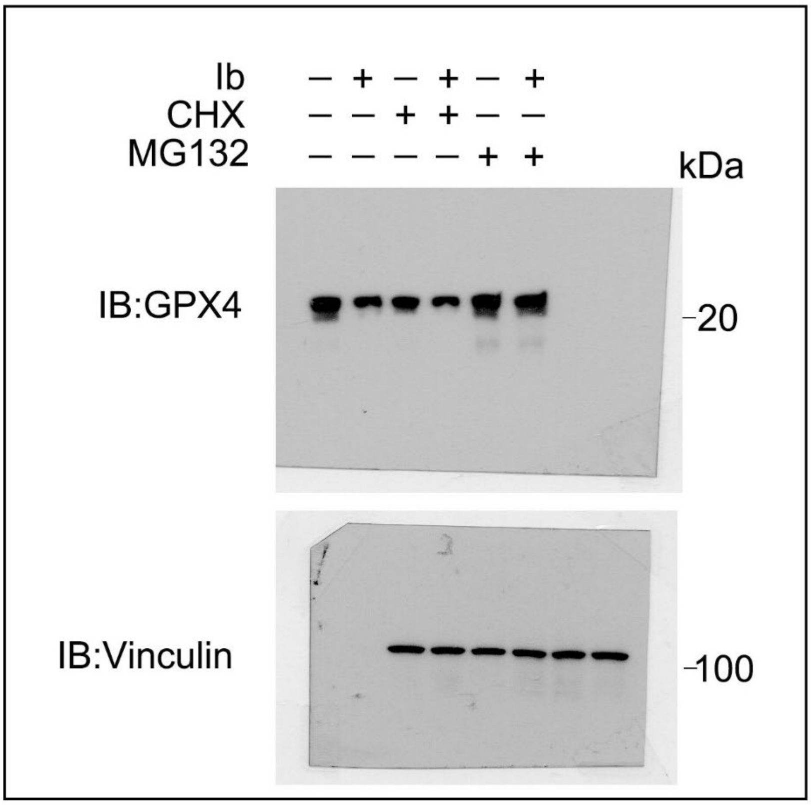

Figure S2. Original images of Figure 5C.

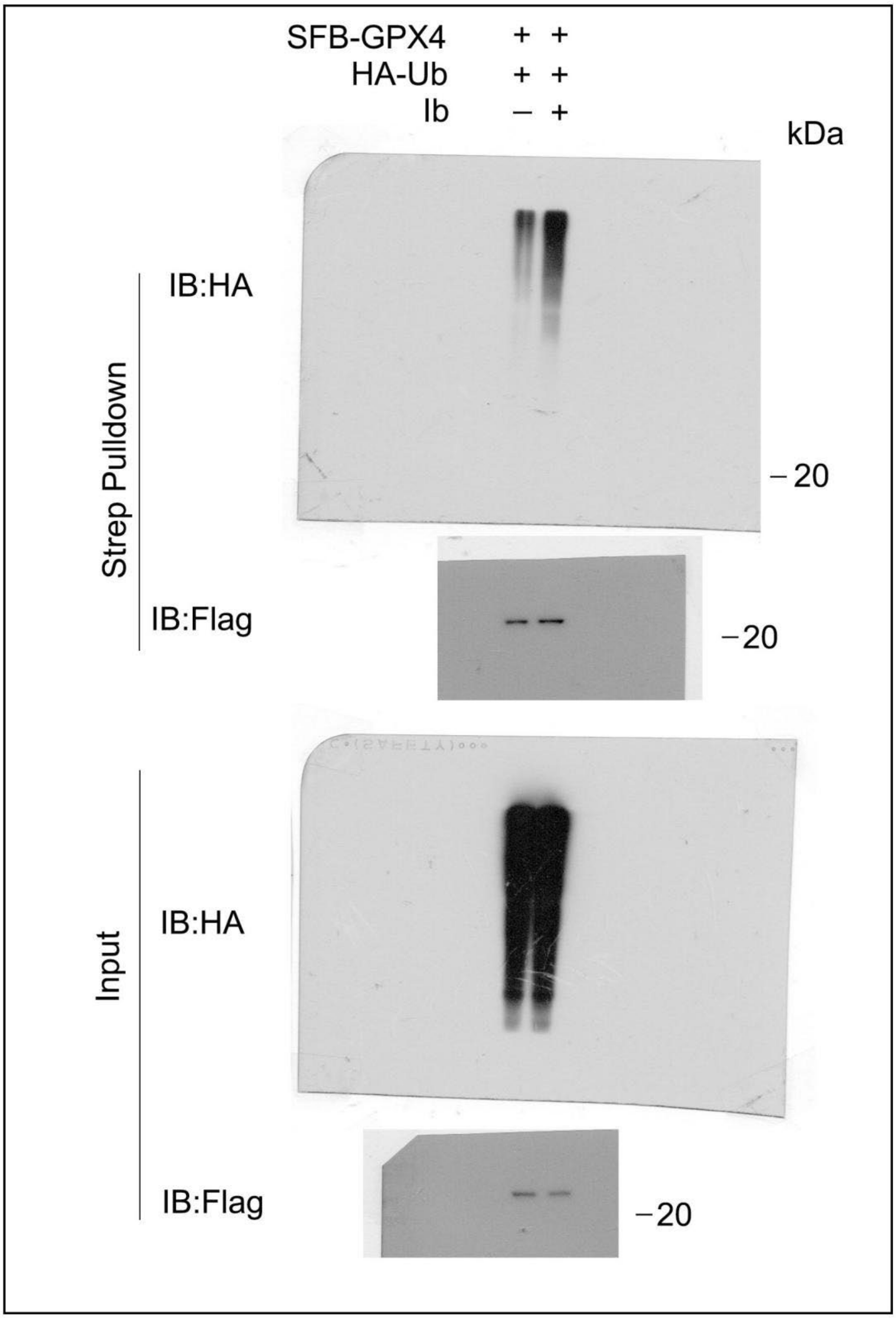

Figure S3. Original images of Figure 5D.
